# Prevalence of multiple sclerosis in an urban population of Sivas province in Turkey

**DOI:** 10.3906/sag-1808-112

**Published:** 2019-02-11

**Authors:** Şeyda Figül GÖKÇE, Burhanettin ÇİĞDEM, Sanem NEMMEZİ KARACA, Aslı BOLAYIR, Özlem KAYIM YILDIZ, Ahmet Suat TOPAKTAŞ, Hatice BALABAN

**Affiliations:** 1 Department of Neurology, Faculty of Medicine, Cumhuriyet University, Sivas Turkey; 2 Department of Family Medicine, Faculty of Medicine, Cumhuriyet University, Sivas Turkey; 3 Department of Neurology, Sivas Medicana Hospital, Sivas Turkey

**Keywords:** Multiple sclerosis, prevalence, Sivas

## Abstract

**Background/aim:**

Multiple sclerosis (MS) is a common neurological disorder that can be a leading cause of nontraumatic disability in several countries. Recent reports have indicated a moderate to high risk of MS in European countries. In this study, we examined the prevalence of MS in a well-defined urban population of provincial center in Sivas Province in Turkey.

**Materials and methods:**

This study sampled all registered residents of urban areas of provincial center in Sivas Province in April 2017 and 2018 January. All the included patients met the McDonald 2010 criteria. Medical records were reviewed, including all available previously acquired magnetic resonance imaging data. All patients were subsequently subjected to neurologic examination to confirm the MS diagnosis.

**Results:**

We identified 21 possible MS patients, with MS diagnosis confirmed in 19. The prevalence of MS was 288 per 100,000 inhabitants.

**Conclusion:**

For future studies, these high ratio results can be used in regional and national comparisons to determine cofactors contributing to the high prevalence of MS in our region and can help health-decision makers to better plan healthcare policies to improve neurological services and awareness about multifaceted clinical presentations of MS.

## 1. Introduction

Multiple sclerosis (MS) is a common neurological disorder that can lead to significant physical disability with more than 30% of MS patients within 20 to 25 years (1). It is the leading cause of nontraumatic neurologic disability in young adults in many countries (2,3). Because of the high prevalence of MS in Caucasians, the epidemiology of MS has been extensively studied and reported primarily for Western countries (4,5). According to a recent review of epidemiologic studies related to MS (2,6), the estimated number of people with MS increased from 2.1 million in 2008 to 2.3 million in 2013, and the estimated female to male ratio of this disorder increased from 1.4:1 to 2.3:1. Most MS patients experience clinical onset between the ages of 20 and 40 years, with a peak age of onset of 24 years in women and 25 years in men (7,8), although clinical onset rarely occurs as early as the first years of life or as late as the seventh decade (8) Several authors have reported a higher frequency of MS among women, especially during pubertal age (9). The prevalence of MS is higher in Northern Europe, America, and South Australia than in Asia and Africa; in addition, there was an increase in the prevalence of MS in other parts of the world, albeit with a low prevalence (10–12). The variation in prevalence depends on several geographical, genetic, and environmental factors (13). 

The occurrence of MS varies geographically by latitude (14). The prevalence of disease is 140/100,000 to 108/100,000 in North America and Europe, and these rates decrease to 2.1/100,000 to 2.2/100,000 in sub-Saharan Africa and East Asia. In the same prevalence registrations, the highest rate, 189/100,000, was found in Sweden, whereas it is 22/100,000 in Albania. MS has a remarkably uneven distribution across the world, with peak prevalence ratios and incidence rates found in the northern hemisphere, where the climate is temperate and income rates are high. In contrast, MS is uncommon in equatorial and tropical countries, particularly among those with low income rates. In addition, in the context of environmental influences, the change in MS prevalence may be related to the population shift from rural to urban areas after industrial development, which causes important changes in environmental factors (15,16). 

As defined by Kurtzke (17), Europe has a high prevalence of MS (≥30/100,000), and it contains more than half of the global population of those diagnosed with MS (18). Nevertheless, much uncertainty remains regarding how the risk of MS varies among European populations. The aim of this study was to systematically review the prevalence and incidence of MS across Europe. However, recent reports have shown increases in the prevalence of MS in Greece, Iran, and other Middle Eastern countries around Turkey. Turkey is considered to have a high risk of MS, with an estimated prevalence of more than 100 cases per 100,000 inhabitants and northern Turkey may be a high-risk area for MS (19). Considering the limitations of the existing data, the prevalence and incidence of MS in the Central Anatolia region of Turkey are unknown.

Relevant epidemiological data may help to have a better understanding of the risk factors of MS and may contribute to the development of new therapeutic modalities. For this purpose, the McDonald 2010 criteria were the most preferred criteria for the diagnostic assessment of MS during the study period (20). Given this situation, MS mortality, incidence, and prevalence data and their evolution over time in Turkey are important for the development of screening and management strategies to reduce the healthcare burden. The prevalence of MS in Sivas Province in Central Anatolia has not been reported previously. With its high altitude and relatively low migration rates, Sivas is an ideal region to be studied as compared with the other regions of Anatolia. The present study was designed to estimate the prevalence of MS using the McDonald 2010 criteria in urban regions of provincial center of Sivas.

## 2. Material and methods

This community-based cross-sectional study was conducted between April 2017 and January 2018, in urban regions of provincial center of Sivas province, located in the eastern part of Turkey. Approval of the study protocol was obtained from the Human Ethics Committee of our University (Registry No. 2016-05/32, dated 27.05.2016). Written informed consent was obtained from the study population.

The distribution and frequency of MS were assessed by estimates of prevalence and incidence. Prevalence refers to the proportion of a population with the disease at or during a specified time, whereas incidence refers to the proportion of new cases arising over a specific period. Prevalence reflects the “stock” of patients, whereas incidence reflects the dynamic dimension of the disease (21).

### 2.1. Study area

Sivas is a city located between the latitudes 38°42ʹ N and 40°16ʹ N, and the longtitudes 35°50ʹ E and 38°14ʹ E. The altitude of the city is 1285 m. Sivas has a dry summer continental climate, with warm and dry summers and cold and snowy winters. The population is homogeneously Turkish, and the middle-poor class dominates socioeconomically. It receives little immigration from other Turkish cities. 

Less exposure to sunlight due to the long winter periods and the style of traditional clothing may be the cause of a high incidence of vitamin D deficiency among the population. The duration of exposure to sunlight in Sivas is below the Turkish average. The population has a habit of staying indoors, and summer vacation in warmer climates is also a rare activity. Consumption of red meat and high carbohydrates is a common nutritional habit as part of their general lifestyle. Along with this eating habit, the lack of consumption of fresh vegetables and fish is also a problem of traditional nutrition. All these characteristics may contribute to the development and progress of MS along with vitamin D deficiency, which may also be a predisposing factor for MS.

### 2.2. Study population 

According to the 2016 census, provincial center of Sivas has a population of 365,135 in the urban region. The study sample was randomly selected from 22 administrative districts using a systematic sampling method. The survey involved 274,704 individuals older than 15 years, and a sample of 6596 (3280 males and 3316 females) was chosen. 

The sample size was calculated using the equation proposed for prevalence research by Naing et al. (22). To calculate the sample size for a prevalence study, the expected prevalence is required. Based on a previous Turkish research (19), the expected prevalence of MS was accepted as 0.001. Therefore, Naing et al.’s (22) equation with the degree of precision (d) = 0.000763 produced a sample size of 6596 in a population size of 274,704. 

In this study, to select the neighborhoods in the study region and the streets in these neighborhoods, researchers benefited from the results of a dissertation thesis including socioeconomic features of urban regions of provincial center of Sivas (7). Neighborhood selection was determined by a cluster sampling method. The streets in each neighborhood were considered to be a cluster, and the streets were selected at a rate of 0.20 and 80%.

### 2.3. Face-to-face survey

Information was collected with a questionnaire through face-to-face interviews with the 6596 respondents or with a household member for those who were illiterate. 

The questionnaire assessed sociodemographic and selected clinical characteristics: sex, age, birthplace, family history, smoking, socioeconomic status, occupation (employed, unemployed, retired, and housewife), age at first diagnosis, disease duration, subtypes of disease (relapsing remitting MS, secondary progressive MS, and primary progressive MS), treatment, and expanded disability status scale (EDSS).

To screen for neurologic findings of MS, we questioned participants about their relapse symptoms and signs of MS. MS relapse was defined by the 2010 McDonald criteria as “patient-reported symptoms or objectively observed signs typical of an acute inflammatory demyelinating event in the CNS (central nervous system), current or historical, with duration of at least 24 hours, in the absence of fever or infection” (20).

The patients’ disability status was evaluated according to the EDSS (6). This scale reflects the rate of disability from 0 to 10 obtained by the evaluation of functional systems; that is, an EDSS score of 4, 7, 8, and 10 represents an ambulatory patient without assistance, unable to walk more than 5 m with assistance, bed and chair dependency, and morbidity due to MS.

### 2.4. Neurologic evaluation

In the second phase of data collection, the clinical aspects of MS in all subjects with clinical and suspected MS findings were evaluated in our neurology outpatient service. 

Patients who were followed up with an MS diagnosis were referred to the clinic and evaluated with clinical, radiologic, and laboratory findings. Participants with previously confirmed MS and newly suspected MS were evaluated according to the 2010 McDonald criteria (20). Patients who did not meet the clinical and radiologic criteria for disease were excluded. 

### 2.5. Statistical analysis

The distribution of characteristics of participants was presented as numbers, percentages, and means. Mann–Whitney and chi-square tests were performed for demographic and selected clinical data of the study population as appropriate. IBM SPSS Statistics (version 23.0) was used for all analyses. A P-value of less than 0.05 was accepted as significant.

## 3. Results

The baseline characteristics of the study population are as shown in Table. During the study period, 21 of the 6595 subjects were found to have MS or MS-related symptoms. Of these 21 patients, 20 had been diagnosed previously and one diagnosed by our research team as a new case; however, in two of the previously diagnosed patients, after neurologic examination and evaluation of previous medical records and laboratory results, we did not confirm MS disease and instead diagnosed cerebrovascular disease and neuromyelitis optica (NMO). Ultimately, we analyzed the clinical data of 19 MS patients. The prevalence of MS was calculated as 288/100,000.

Of 18 MS patients, 9 were followed by our neurology service, 5 were diagnosed in our neurology service but followed in another health institution, 1 was diagnosed and followed by another health institution in other Turkish cities, and 3 did not complete follow-up.

**Table 1:** Selected demographic and clinical data of the study population.

	Female (n = 11)	Male (n = 8)
Age (y)	40 (23–70)	38 (23–47)
Birth place Sivas Other Turkish city Other country	9 (81.8%) 1 (9.1%) 1 (9.1%)	8 (100%)
Family history Yes No	3 (27.3) 8 (72.7%)	1 (12.5%) 7 (87.5%)
Smoking Current Never Past	2 (18.2%) 9 (81.8%)	5 (62.5%)^a^ 2 (25.0%) 1 (12.5%)
Socioeconomic status Low Moderate	4 (36.4%) 7 (63.6%)	2 (25.0%) 6 (75.0%)
Occupation Employed Retired Unemployed Housewife	3(27.3%) 2 (18.2%) 6 (54.5%)	4 (50.0%) 3 (37.5%) 1 (12.5%)
Age at first diagnosis (y)	28 (19–43)	26 (18–35)
Disease duration (y)	8 (1–35)	11.5 (1–20)
Subtypes of disease RRMS SPRS PPMS	7 (63.6%) 3 (27.3%) 1 (9.1 %)	6 (75.0%) 1 (12.5%) 1 (12.5%)
Treatment Yes No	10 (90.9%) 1 (9.1%)	7 (87.5%) 1 (12.5%)
EDSS	1 (0.5–8)	2.5 (0.5–8)

Data are presented as median (min–max) or number (percentage). aP < 0.05 vs. female. Other variables were found to be similar (P > 0.05). Abbreviations: RRMS, relapsing-remitting multiple sclerosis; SPMS, secondary progressive multiple sclerosis; PPMS, primary progressive multiple sclerosis; EDSS, expanded disability status scale.

The median ages of the female and male patients were similar (P > 0.05). The birthplace of nearly all the study population was Sivas. Among the study population, 4 (21%) patients had a family history of MS. The active smoking rate was significantly higher in male patients (62.5% vs. 18.2%; P < 0.05). The socioeconomic status and subtypes of disease of female and male patients were comparable (P > 0.05). Most of the female patients were housewives, and half of the male patients were actively working. Of both female and male patients, the ages at first diagnosis, disease duration, and EDSS values were found to be similar (P > 0.05). Nearly 90% of the study population was receiving treatment for MS.

## 4. Discussion

In the current survey, we screened 6596 subjects aged 15 years and older in the urban areas of provincial center of Sivas, which is in a high-altitude region of eastern Anatolia. We confirmed the diagnosis of MS in 19 subjects, and the MS prevalence was calculated as 288/100,000. However, in the first phase of the study, two patients were found as under MS follow-up, and in the neurologic evaluation phase, we could not confirm their diagnosis as MS. Their diagnoses were changed to cerebrovascular disease and NMO.

In the current study, there were more female than male MS subjects (11 vs. 8). The ages of the female and male participants were similar. All subjects were predominantly from Sivas. This is an important point that increases the reliability of our data about the MS prevalence in Sivas. A closed lifestyle and limited migration as local regional features may be predisposing genetic factors for MS. Although there were small case numbers, family history was more pronounced in female subjects (3 vs. 1 subject). Current smoking in male subjects was meaningful compared with female subjects. All patients were mainly from a moderate socioeconomic class. Being a housewife and being employed were meaningful in female and male subjects, respectively. Age at first diagnosis was in accordance with other studies in Turkey and European countries (23,24). There was no meaningful difference between female and male subjects in terms of disease duration, subtype of disease, use of treatment, or EDSS value. These findings, overall, are in accordance with our experience in MS diagnosis, management, and follow-up. Although a few Turkish studies exist on the prevalence of MS and related factors influencing the disease, it is obviously necessary to carry out further regional and countrywide studies and differentiate the influence of factors, including geographic, socioeconomic, and lifestyle variables.

Knowledge about distribution of MS patients in the country may cause an important impact on the development of new strategies to decrease the burden of this chronic condition on the healthcare system with rational use of national resources. At the national level, a heterogeneous distribution of cases over a territory requires adequate distribution of healthcare resources. Moreover, determination of the geographical variation in MS prevalence would lead to novel ways that research can further explore the spatial or environmental conditions affecting healthcare for chronic neurologic diseases (25,26). The MS prevalence of 288/100,000 found in our study is much higher those than reported in studies conducted in other regions or cities in Turkey at various dates. In a prevalence study involving six cities in the Central Black Sea Region of Turkey, the MS prevalence was found to be 43.2/100,000 in 2017. In that study, the MS prevalence of 46.5/100,000 was reported in Samsun Province. Those data including 1787 MS patients were obtained from hospital records of Samsun, Sinop, Ordu, Amasya, Tokat, and Çorum Provinces over a 10-year period (23). In a door-to-door epidemiological field study conducted by Alp et al. (27), the prevalence of MS in Kars Province of northeastern Turkey was reported as 69/100,000. In another Turkish study in three cities of the Black Sea region, conducted via door-to-door interviews in rural areas, the MS prevalence was 61/100,000, 41/100,000, and 53/100,000 in Kandıra, Gevye, and Erbaa counties, respectively (24). In a prevalence study conducted in İstanbul in 2006 by the same researchers, the MS prevalence was found to be 101.4/100,000 (19). 

In studies conducted around the world, the prevalence of MS has been reported to be lower in Siberia, European Russia, Macedonia, Bulgaria, Greece, China, Japan, and the Middle East (5). The lowest rate in the world is in Malta (4/100,000) (28). A higher frequency of MS is consistently found in Nordic countries (28–31), northern parts of Western Europe, and North America, whereas lower frequencies of MS have been reported in Asia, the Middle East, and Africa (17,32–34). The highest prevalence rates found in studies conducted in other European cities are 203/100,000 in the United Kingdom (35), 203/100,000 in Norway (34), 215/100,000 in Sweden (36), 175/100,000 in Germany (37), 170/100,000 in France (38), and 290/100,000 in Northwest Ireland (39). The highest reported rate in the United States is 192/100,000 (40). The highest reported rate in the world was found in Orkney, Scotland, at 410/100,000 (41). In recent decades, many studies have reported an increasing prevalence of MS among various parts of the world, including Sweden, Finland, Japan, and Iran (42–45), whereas other studies found a more stable prevalence (46). The reason for the apparent increase in some studies is not known but may be a result of improvements in diagnostic imaging or an increased focus on MS in association with increased availability of effective treatment (47).

Multiple factors have the potential to contribute to the increase in MS prevalence (48–51). First, an actual increase, due to underdetermined factors, in the frequency of MS is possible. Second, improvements in diagnosis and the incorporation of magnetic resonance imaging (MRI) and new diagnostic criteria may result in earlier diagnosis and improved detection of cases that are clinically milder (52). Third, case detection might be increased due to general improvement in healthcare services and awareness of the condition. Finally, an increase in long-term survival might be explained by improved general medical care and the possible beneficial effects of disease-modifying therapy (53).

Prevalence studies conducted thus far have shown that MS prevalence differs by geography and increases especially as the distance from the equator increases. However, rates below the estimates were also observed in places far from the equator, where the prevalence was expected to be high. This was attributed to the fact that the habit of eating seafood is protective against the disease (54). From this point of view, it seems that intake of vitamin D from sunlight or via the oral route is decreasing the frequency of disease (55). There are also places in the world where the prevalence rates are significantly higher than the general distribution in different regions of this country, especially in Europe and America. This difference is reported to be due to the local genetic characteristics of the population, rather than environmental factors such as nutrition and infection. A detailed investigation of the environmental factors and genetic characteristics of etiology in residential areas with high prevalence will provide a better understanding of the etiopathogenesis of the disease.

In cities with less migration of people, marriage within the same community may modify the genetic basis of the population for the development of some chronic diseases, including MS. Even though there is a high amount of sunlight in the study area, both traditional habit and dressing for protection from cold as well as a preference for an indoor environment in daily life can create a deficiency of vitamin D, which may be an environmental factor that plays a role in MS pathology. In addition, the fact that vitamin D is not taken with food (in addition to a preference for vegetables, seafood, and fresh fruit instead of red meat, saturated fat, and carbohydrate) may also contribute to the high prevalence of MS (55,56).

In summary, the number of studies on the prevalence of MS disease in Turkey is very low. The rates found in these studies are very low compared with the results of this study. The results obtained in Sivas Provincial Center, which is a Central Anatolian city, are the highest ever reported in Turkey. This result may depend on the closed genetic characteristic of the region but may also be a result of environmental factors, nutritional characteristics, and lifestyle-related characteristics. The values obtained in this study will be useful in planning further studies as well as in elucidating the etiopathogenesis of the disease and determining appropriate treatment strategies. Healthcare and support services have improved compared with 2008 (e.g., increased numbers of neurologists, MRI machines).

Regarding the methodological strengths and limitations of the current study, the cross-sectional design has limitations in terms of causal interpretation of clinical data. In addition, it does not account for the change of prevalence because of time. This study has significant strengths, including neurologic evaluation performed in the same outpatient service with a similar setup, use of only the McDonald 2010 criteria for the final diagnosis of MS patients, a small rate of migration to Sivas, and the opportunity to enroll subjects with the same genetic and environmental background. 

Our study was a population-based, cross-sectional study and the first of its kind to provide a considerably accurate estimate of the prevalence of MS in Sivas. To the best of our knowledge, there are no previously reported data on the prevalence in our region. This study was conducted because of the limited number of studies on the prevalence of MS in Turkey, the different prevalence rates across regions, and the lack of studies focusing on the Central Anatolian region. During the study period, we found 2 subjects with a wrong diagnosis of MS, suggesting that in Sivas, there is an important need to increase neurologic healthcare and health workers’ awareness of MS and other chronic neurologic diseases. Our results can be used in future studies of regional and national comparisons to determine cofactors that contribute to the high prevalence of MS in our region and can also help health-decision makers to better plan healthcare policies to improve neurologic services and awareness about the multifaceted nature of MS.
